# 4,4′-Di-3-pyridyl-2,2′-dithio­dipyrimidine

**DOI:** 10.1107/S1600536809016869

**Published:** 2009-05-14

**Authors:** Jun-Feng Ji, Lei Li, Hai-Bin Zhu

**Affiliations:** a School of Chemistry and Chemical Engineering, Southeast University, Nanjing 211189, People’s Republic of China

## Abstract

The asymmetric unit of the title compound, C_18_H_12_N_6_S_2_, contains one half-mol­ecule situated on a twofold rotational axis that passes through the mid-point of the S—S bond. In the mol­ecule, the C—S—S—C torsion angle is 81.33 (7)°. The crystal packing exhibits no significantly short inter­molecular contacts.

## Related literature

For general background to heterocyclic disulfides, see Horikoshi & Mochida (2006 ▶). For related crystal structures, see: Higashi *et al.* (1978 ▶); Tabellion *et al.* (2001 ▶).
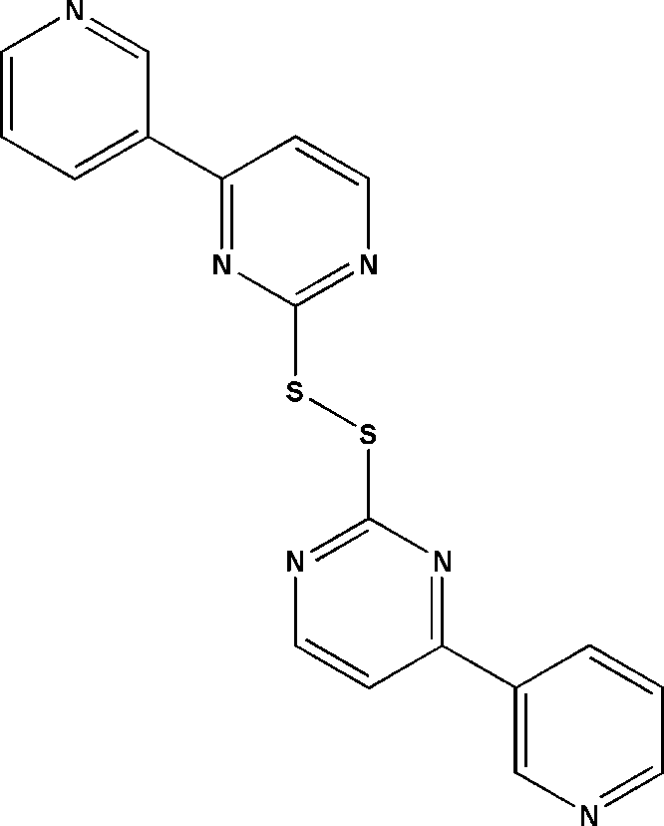


## Experimental

### 

#### Crystal data

C_18_H_12_N_6_S_2_
*M* *_r_* = 376.48
Monoclinic, 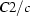

*a* = 19.480 (3) Å
*b* = 5.4192 (9) Å
*c* = 17.979 (3) Å
β = 115.034 (2)°
*V* = 1719.6 (5) Å^3^
*Z* = 4
Mo *K*α radiation
μ = 0.33 mm^−1^
*T* = 298 K
0.12 × 0.11 × 0.09 mm

#### Data collection

Bruker APEXII CCD area-detector diffractometer
Absorption correction: multi-scan (*SADABS*; Bruker, 2001 ▶) *T*_min_ = 0.884, *T*_max_ = 0.920 (expected range = 0.933–0.971)
5331 measured reflections
2091 independent reflections
1590 reflections with *I* > 2σ(*I*)
*R*_int_ = 0.054

#### Refinement

*R*[*F* ^2^ > 2σ(*F* ^2^)] = 0.039
*wR*(*F* ^2^) = 0.106
*S* = 1.07
2091 reflections
118 parameters
H-atom parameters constrained
Δρ_max_ = 0.20 e Å^−3^
Δρ_min_ = −0.25 e Å^−3^

### 

Data collection: *APEX2* (Bruker, 2007 ▶); cell refinement: *SAINT-Plus* (Bruker, 2007 ▶); data reduction: *SAINT-Plus*; program(s) used to solve structure: *SHELXS97* (Sheldrick, 2008 ▶); program(s) used to refine structure: *SHELXL97* (Sheldrick, 2008 ▶); molecular graphics: *SHELXTL* (Sheldrick, 2008 ▶); software used to prepare material for publication: *SHELXTL*.

## Supplementary Material

Crystal structure: contains datablocks I, New_Global_Publ_Block. DOI: 10.1107/S1600536809016869/cv2555sup1.cif

Structure factors: contains datablocks I. DOI: 10.1107/S1600536809016869/cv2555Isup2.hkl

Additional supplementary materials:  crystallographic information; 3D view; checkCIF report

## Figures and Tables

**Fig. 1.**
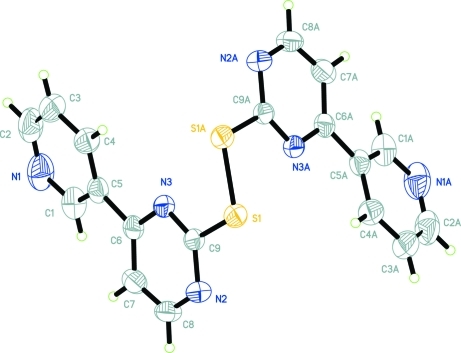
The molecular structure of the title compound showing the atomic numbering and 40% probability displacement ellipsoids [symmetry code: (A) -x, y, 1/2 - z].

## supplementary crystallographic information

### Comment

Heterocyclic disulfide ligands have attracted considerable attention due to its conformationally defined torison angle and axial chirality (Horikoshi & Mochida, 2006). Herein, we report the molecular structure of the title compound (I) - the newly synthesized disulfide ligand.

In (I) (Fig. 1), the dihedral angle between the pyrimidinyl and pyrdinyl rings is 17.62 (6)°. The C—S—S—C torsion angle of 81.33 (7)° and S—S bond length of 2.0148 (8) Å are comparable to those of typical aromatic disulfides (Higashi *et al.*, 1978; Tabellion *et al.*, 2001).

### Experimental

A solution of SO_2_Cl_2_ (0.5 mL) in CH_2_Cl_2 _(20 ml) was added dropwise into the suspension containing 4-(pyridin-3-yl)pyrimidine-2-thiol (1.89 g) and 30 ml of CH_2_Cl_2_.Upon addition, the mixture was stirred at room temperature for 30 min. The solid was collected by filtration and dissolved into 30 ml of H_2_O. The solution PH was adjusted into the range of 8–9 to give white precipitates. Single crystals suitable for X-ray diffraction analysis were obtained by slow evaporation of the CH_2_Cl_2_ solution of the title compound.

### Refinement

All H atoms were positioned geometrically and allowed to ride on their parent atoms, with C—H = 0.93 Å and *U*iso(H) = 1.2*U*eq(C).

### Crystal data

**Table:**

C_18_H_12_N_6_S_2_	*F*(000) = 776
*M**_r_* = 376.48	*D*_x_ = 1.454 Mg m^−^^3^
Monoclinic, *C*2/*c*	Mo *K*α radiation, λ = 0.71073 Å
Hall symbol: -C 2yc	Cell parameters from 2091 reflections
*a* = 19.480 (3) Å	θ = 2.3–25.5°
*b* = 5.4192 (9) Å	µ = 0.33 mm^−^^1^
*c* = 17.979 (3) Å	*T* = 298 K
β = 115.034 (2)°	Block, yellow
*V* = 1719.6 (5) Å^3^	0.12 × 0.11 × 0.09 mm
*Z* = 4	

### Data collection

**Table:**

Bruker APEXII CCD area-detector diffractometer	2091 independent reflections
Radiation source: fine-focus sealed tube	1590 reflections with *I* > 2σ(*I*)
graphite	*R*_int_ = 0.054
φ and ω scans	θ_max_ = 28.2°, θ_min_ = 2.3°
Absorption correction: multi-scan (*SADABS*; Bruker, 2001)	*h* = −15→25
*T*_min_ = 0.884, *T*_max_ = 0.920	*k* = −7→6
5331 measured reflections	*l* = −23→21

### Refinement

**Table:**

Refinement on *F*^2^	Primary atom site location: structure-invariant direct methods
Least-squares matrix: full	Secondary atom site location: difference Fourier map
*R*[*F*^2^ > 2σ(*F*^2^)] = 0.039	Hydrogen site location: inferred from neighbouring sites
*wR*(*F*^2^) = 0.106	H-atom parameters constrained
*S* = 1.07	*w* = 1/[σ^2^(*F*_o_^2^) + (0.051*P*)^2^] where *P* = (*F*_o_^2^ + 2*F*_c_^2^)/3
2091 reflections	(Δ/σ)_max_ < 0.001
118 parameters	Δρ_max_ = 0.20 e Å^−^^3^
0 restraints	Δρ_min_ = −0.25 e Å^−^^3^

### Special details

**Table:**

Geometry. All e.s.d.'s (except the e.s.d. in the dihedral angle between two l.s. planes) are estimated using the full covariance matrix. The cell e.s.d.'s are taken into account individually in the estimation of e.s.d.'s in distances, angles and torsion angles; correlations between e.s.d.'s in cell parameters are only used when they are defined by crystal symmetry. An approximate (isotropic) treatment of cell e.s.d.'s is used for estimating e.s.d.'s involving l.s. planes.
Refinement. Refinement of *F*^2^ against ALL reflections. The weighted *R*-factor *wR* and goodness of fit *S* are based on *F*^2^, conventional *R*-factors *R* are based on *F*, with *F* set to zero for negative *F*^2^. The threshold expression of *F*^2^ > σ(*F*^2^) is used only for calculating *R*-factors(gt) *etc*. and is not relevant to the choice of reflections for refinement. *R*-factors based on *F*^2^ are statistically about twice as large as those based on *F*, and *R*- factors based on ALL data will be even larger.

### Fractional atomic coordinates and isotropic or equivalent isotropic displacement parameters (Å^2^)

**Table:**

	*x*	*y*	*z*	*U*_iso_*/*U*_eq_	
S1	0.03745 (2)	0.82831 (7)	0.22489 (2)	0.05022 (17)	
N3	0.09221 (7)	0.4611 (2)	0.33598 (7)	0.0431 (3)	
C5	0.13449 (9)	0.1539 (3)	0.44158 (9)	0.0494 (4)	
C6	0.14360 (8)	0.2851 (3)	0.37363 (9)	0.0449 (4)	
N2	0.15519 (8)	0.5576 (3)	0.25070 (8)	0.0560 (4)	
C9	0.10152 (8)	0.5858 (3)	0.27763 (8)	0.0436 (3)	
C7	0.20210 (9)	0.2385 (3)	0.35106 (10)	0.0549 (4)	
H7A	0.2380	0.1168	0.3769	0.066*	
C8	0.20481 (10)	0.3797 (3)	0.28883 (11)	0.0599 (5)	
H8A	0.2434	0.3493	0.2725	0.072*	
C4	0.09033 (11)	0.2523 (4)	0.47734 (10)	0.0617 (5)	
H4A	0.0642	0.3995	0.4582	0.074*	
C1	0.17081 (12)	−0.0663 (3)	0.47231 (10)	0.0668 (5)	
H1A	0.2002	−0.1334	0.4479	0.080*	
N1	0.16688 (12)	−0.1900 (3)	0.53450 (11)	0.0827 (6)	
C3	0.08571 (12)	0.1278 (5)	0.54219 (11)	0.0771 (6)	
H3B	0.0569	0.1906	0.5679	0.092*	
C2	0.12428 (14)	−0.0895 (5)	0.56778 (12)	0.0858 (7)	
H2B	0.1204	−0.1720	0.6112	0.103*	

### Atomic displacement parameters (Å^2^)

**Table:**

	*U*^11^	*U*^22^	*U*^33^	*U*^12^	*U*^13^	*U*^23^
S1	0.0489 (3)	0.0576 (3)	0.0448 (2)	−0.00386 (18)	0.02041 (19)	0.00495 (17)
N3	0.0412 (7)	0.0496 (7)	0.0378 (6)	−0.0045 (6)	0.0159 (5)	−0.0041 (5)
C5	0.0477 (9)	0.0511 (9)	0.0405 (8)	−0.0095 (7)	0.0101 (7)	−0.0034 (6)
C6	0.0417 (8)	0.0475 (8)	0.0394 (8)	−0.0074 (7)	0.0113 (7)	−0.0102 (6)
N2	0.0466 (8)	0.0760 (10)	0.0524 (8)	−0.0031 (7)	0.0278 (7)	−0.0019 (7)
C9	0.0389 (7)	0.0533 (8)	0.0375 (7)	−0.0080 (7)	0.0150 (6)	−0.0080 (6)
C7	0.0427 (9)	0.0596 (9)	0.0578 (10)	0.0013 (8)	0.0170 (8)	−0.0079 (8)
C8	0.0454 (9)	0.0795 (12)	0.0626 (10)	−0.0025 (9)	0.0304 (8)	−0.0114 (9)
C4	0.0586 (11)	0.0721 (11)	0.0550 (10)	−0.0076 (9)	0.0246 (9)	0.0058 (8)
C1	0.0716 (12)	0.0599 (11)	0.0551 (10)	−0.0021 (10)	0.0133 (9)	0.0008 (9)
N1	0.0928 (14)	0.0723 (11)	0.0631 (10)	−0.0090 (10)	0.0137 (10)	0.0172 (8)
C3	0.0734 (14)	0.1040 (16)	0.0558 (11)	−0.0123 (12)	0.0292 (10)	0.0095 (10)
C2	0.0838 (16)	0.1026 (17)	0.0541 (11)	−0.0319 (14)	0.0129 (11)	0.0196 (11)

### Geometric parameters (Å, °)

**Table:**

S1—C9	1.7840 (16)	C7—H7A	0.9300
S1—S1^i^	2.0148 (8)	C8—H8A	0.9300
N3—C9	1.3233 (17)	C4—C3	1.383 (2)
N3—C6	1.3402 (19)	C4—H4A	0.9300
C5—C1	1.378 (2)	C1—N1	1.333 (2)
C5—C4	1.380 (2)	C1—H1A	0.9300
C5—C6	1.488 (2)	N1—C2	1.328 (3)
C6—C7	1.385 (2)	C3—C2	1.367 (3)
N2—C9	1.334 (2)	C3—H3B	0.9300
N2—C8	1.332 (2)	C2—H2B	0.9300
C7—C8	1.375 (2)		
			
C9—S1—S1^i^	103.78 (5)	N2—C8—H8A	118.2
C9—N3—C6	116.12 (13)	C7—C8—H8A	118.2
C1—C5—C4	117.59 (17)	C5—C4—C3	118.7 (2)
C1—C5—C6	121.58 (16)	C5—C4—H4A	120.7
C4—C5—C6	120.81 (15)	C3—C4—H4A	120.7
N3—C6—C7	120.87 (14)	N1—C1—C5	124.72 (19)
N3—C6—C5	115.57 (13)	N1—C1—H1A	117.6
C7—C6—C5	123.53 (15)	C5—C1—H1A	117.6
C9—N2—C8	113.93 (13)	C2—N1—C1	116.12 (18)
N3—C9—N2	128.37 (15)	C2—C3—C4	118.8 (2)
N3—C9—S1	119.86 (11)	C2—C3—H3B	120.6
N2—C9—S1	111.77 (11)	C4—C3—H3B	120.6
C8—C7—C6	117.17 (16)	N1—C2—C3	124.10 (19)
C8—C7—H7A	121.4	N1—C2—H2B	118.0
C6—C7—H7A	121.4	C3—C2—H2B	118.0
N2—C8—C7	123.54 (15)		

Symmetry codes: (i) −*x*, *y*, −*z*+1/2.
